# Strategies to Estimate Prevalence of SARS-CoV-2 Antibodies in a Texas Vulnerable Population: Results From Phase I of the Texas Coronavirus Antibody Response Survey

**DOI:** 10.3389/fpubh.2021.753487

**Published:** 2021-12-14

**Authors:** Melissa A. Valerio-Shewmaker, Stacia DeSantis, Michael Swartz, Ashraf Yaseen, Michael O. Gonzalez, Harold W. III Kohl, Steven H. Kelder, Sarah E. Messiah, Kimberly A. Aguillard, Camille Breaux, Leqing Wu, Jennifer Shuford, Stephen Pont, David Lakey, Eric Boerwinkle

**Affiliations:** ^1^School of Public Health, University of Texas Health Science Center, Brownsville, TX, United States; ^2^School of Public Health, University of Texas Health Science Center, Austin, TX, United States; ^3^Texas Department of State Health Services, Austin, TX, United States; ^4^University of Texas System, Population Health, Austin, TX, United States; ^5^School of Public Health, University of Texas Health Science Center, Dallas, TX, United States

**Keywords:** antibodies, COVID-19, health disparities, population methods, Federally Qualified Health Center (FQHC)

## Abstract

**Introduction:** Severe acute respiratory syndrome coronavirus 2 (SARS-CoV-2) infection and immunity remains uncertain in populations. The state of Texas ranks 2nd in infection with over 2.71 million cases and has seen a disproportionate rate of death across the state. The Texas CARES project was funded by the state of Texas to estimate the prevalence of SARS-CoV-2 antibody status in children and adults. Identifying strategies to understand natural as well as vaccine induced antibody response to COVID-19 is critical.

**Materials and Methods:** The Texas CARES (Texas Coronavirus Antibody Response Survey) is an ongoing prospective population-based convenience sample from the Texas general population that commenced in October 2020. Volunteer participants are recruited across the state to participate in a 3-time point data collection Texas CARES to assess antibody response over time. We use the Roche Elecsys® Anti-SARS-CoV-2 Immunoassay to determine SARS-CoV-2 antibody status.

**Results:** The crude antibody positivity prevalence in Phase I was 26.1% (80/307). The fully adjusted seroprevalence of the sample was 31.5%. Specifically, 41.1% of males and 21.9% of females were seropositive. For age categories, 33.5% of those 18–34; 24.4% of those 35–44; 33.2% of those 45–54; and 32.8% of those 55+ were seropositive. In this sample, 42.2% (89/211) of those negative for the antibody test reported having had a COVID-19 test.

**Conclusions:** In this survey we enrolled and analyzed data for 307 participants, demonstrating a high survey and antibody test completion rate, and ability to implement a questionnaire and SARS-CoV-2 antibody testing within clinical settings. We were also able to determine our capability to estimate the cross-sectional seroprevalence within Texas's federally qualified community centers (FQHCs). The crude positivity prevalence for SARS-CoV-2 antibodies in this sample was 26.1% indicating potentially high exposure to COVID-19 for clinic employees and patients. Data will also allow us to understand sex, age and chronic illness variation in seroprevalence by natural and vaccine induced. These methods are being used to guide the completion of a large longitudinal survey in the state of Texas with implications for practice and population health.

## Introduction

Since January 2020, the Centers for Disease Control (CDC) has recommended county and state level reporting of all laboratory-confirmed cases of SARS-CoV-2 infection (1). However, reported cases likely represent only a fraction of SARS-CoV-2 infections across the United States, as a still unknown proportion of cases are mild or asymptomatic (2–5), especially in young adults or children (5–10). Other challenges for SARS-CoV-2 surveillance include under-reported cases due to local health department capacity, delays in recording of testing and various methods of test reporting (2–4, 11). Also missing is our understanding of the human response to natural and vaccine induced antibodies over time. Understanding of who is To obtain a more accurate representation of infection, many states and countries have turned to estimating SARS-CoV-2 seroprevalence from blood antibody assays allowing for an estimate of the prevalence of the human antibody response (11–14).

Published data from the COVID-19 first and second wave indicate infections rates vary widely among different populations and geographic regions within a state (11). Highly exposed populations include front line essential workers such as health care workers, teachers and educational staff, and those working in service, business, and retail, including grocery stores (4). Furthermore, ethnic minorities are at higher risk of contracting COVID-19 (7, 8) as are vulnerable populations such as those without health insurance, people experiencing homelessness, or those with pre-existing conditions such as type 2 diabetes, hypertension and asthma (15–17). Black and Latino communities have been especially hard hit by COVID-19 (18–20); for example, in a New York State Department of Health (NYSDOH) convenience sample of 15,000 New Yorkers *employed* at 99 grocery stores across 26 counties representing 87.3% of the state's population found an adjusted seroprevalence ranging from 8.1% in non-Hispanic whites to 29.2% in Latinos, with an overall seroprevalence in New York City of 22.7%, vs. a state-wide prevalence of only 8.9%. Other large seroprevalence studies are being conducted in California, Colorado, Georgia and Ohio (11, 18, 20).

Texas is the second largest state in the country and has a diverse population over 28,250,000 and a majority minority, with ~40% of residents identifying as Hispanic ethnicity, 12% as Black non-Hispanic and 7% other ethnicity. More than 34% of Texans live below 200% of federal poverty level (FPL). Texas is geographically diverse with ~85% of residents living in urban centers with vast rural areas requiring over 1 h of travel to regional hospital systems (21). Several areas of Texas have seen a high incidence of confirmed coronavirus disease (COVID-19) cases across two surges (July and December), including Dallas, Harris, Nueces, Cameron and Hidalgo counties. Furthermore, the prevalence of confirmed COVID-19 varies significantly across the state and by employment industry. For example, higher proportions of confirmed tests have been observed in underserved urban areas such as Dallas and Houston (22, 23) and in areas with a high prevalence of vulnerable or Latino populations, such as San Antonio and McAllen, and in areas with multi-generational households, where viral transmission may be increased due to higher household density and with varied age groups within one household. Additionally, disparate burden of infection in rural areas with immigration detention centers (Willacy Co.) and meatpacking plants in the Texas Panhandle region (23).

To ascertain estimate exposure to SARS-CoV-2 in the state of Texas, and to obtain an understanding of exposure across Texas, the Texas Coronavirus Antibody Research survey (Texas CARES) was designed as a longitudinal antibody surveillance study using a convenience sample approach from among highly exposed populations. This is a unique project as it purposely uses a voluntary approach to reach communities across Texas to explore both natural and vaccine induced antibody response and its duration. Phase I of Texas CARES was designed to identify the feasibility of partnering and reaching vulnerable patients at Federally Qualified Health Centers (FQHCs) and to estimate seroprevalence of the 319 participants in this phase. There are currently 73 FQHCs serving patients in Texas, operating more than 500 sites and two FQHC lookalikes which offer FQHC-like services. The FQHCs are located across 126 counties and serve over 400,000 Medicaid patients, 28% of all FQHC patients, with 1,426,019 million patients served annually and over 5,300,000 patient visits annually (24). We report here our Phase I sub-study of seroprevalence in a sample of 319 adults enrolling at three FQHC sites in Texas. Allowing us to better identify and understand natural and vaccine responses in vulnerable and underserved populations for which mitigation efforts may not be afforded, understanding their response over time will allow us to better prepare future public health responses.

## Methods

All study protocols were reviewed and approved by the University of Texas Health Science Center Houston Institutional Review Board prior to any data collection. The Texas CARES program is a partnership with Texas Department of State Health Services and the University of Texas System with a statewide laboratory partner, Clinical Pathology Laboratories (CPL). The Texas Association of Community Health Centers (TACHC) partnered with us to introduce the program to FQHC sites. In total 40 or more FQHCs will be enrolled in the program over time.

### Study Population

The Phase I sub study of 307 participants presenting at or working at three FQHCs was performed as part of the larger Texas CARES study. The larger study aims to enroll participants from four populations across the state of Texas; pediatric school children 5–17 years of age, FQHC or community clinic patients, kindergarten to −12th grade educators and allied staff and Texas workforce employees who will be tested for SARS-CoV-2 antibodies at three points over a 6–12 month period. The Texas CARES uses a convenience sample of Texans representing the four populations across the state. The next phases of Texas CARES have expanded to recruitment of all Texans across industries with an emphasis on teachers, education setting employees, universities and community residents. We have also begun collecting natural and vaccine induced antibody response in the total Texas CARES program population.

For Phase I, on the day patients presented for their healthcare appointments, an FQHC healthcare team member offered adults 18–80 years of age literature on the Texas CARES and the Roche Elecsys^®^ Anti-SARS-CoV-2 test (2021 Roche Diagnostics, North America), and invited them or their children (5–17 years of age) to enroll in the study. Participation was limited to two representatives from the same household between 5 and 80 years of age. Enrollment required contact information, demographic characteristics and informed consent for three blood draws over 6–12 months. Patients who consented to enroll in Texas CARES were provided a questionnaire collecting demographic information, employment, baseline medical conditions and comorbidities, prior COVID-19 tests and diagnoses, physician diagnosis of COVID-19 and other high-risk chronic illnesses such as type 2 diabetes, asthma and hypertension, COVID-19 symptoms and severity, and COVID-19 behavioral health (25).

#### SARS Cov-2 Antibody Assay Roche Diagnostics

The primary outcome was a positive antibody assay qualitatively assessed using the Roche Elecsys® Anti-SARS-CoV-2 Immunoassay developed to detect antibodies to SARS-Cov-2. The Anti-SARS-CoV-2 Immunoassay has received Emergency Use Authorization (EUA) by the U.S. Food and Drug Administration. The Elecsys® Anti-SARS-CoV-2 Immunoassay detects high-affinity antibodies to SARS-CoV-2 using a modified recombinant protein representing the nucleocapsid (N) antigen for the determination of SARS-CoV-2 antibodies. The test has a published sensitivity of 99.82% sensitivity (95% CI: 99.69–99.91) and 99.91% specificity in diagnostic specimens (*n* = 2,861) (26). The qualitative test results are provided to participants by text to ensure receipt, follow up by phone or email is made as needed to reach the vulnerable population.

#### Questionnaire

A programmed questionnaire was designed to be completed in 10–15 min to capture demographic and clinical characteristics including BMI, comorbidities, prior COVID-19 virus testing, positivity, COVID-19 symptoms, previous antibody testing and mental health during the pandemic (27). To help ensure validity, wherever possible, all questionnaire headers, questions, and response formats were harmonized to the PhenX Toolkit for COVID-19 and the BRFSS questionnaires. PhenX Toolkit items were reviewed for appropriateness, BRFSS and U.S. Census race/ethnicity questions were used. All study materials, including the questionnaire, were available in both English and Spanish.

It was decided *a priori* that a survey weblink would be emailed and texted to those completing fewer than 50% of questions at their medical visit (28, 29). Those who did not respond by completing the survey received a phone call from a team member to collect the survey data. The survey completion percentage in our phase I study of 307 participants prior to the phone call was 96%, which is an indicator both of good validity and construction of our protocols.

### Primary Outcomes and Statistical Analyses

The primary outcomes of Phase I included: (1) feasibility of implementation of the questionnaire and SARS-CoV-2 testing in a highly vulnerable population including children, and (2) estimation of Texas demographic and assay-adjusted cross-sectional seroprevalence based on antibody test results in these participants. The descriptive statistics are reported.

#### Prevalence Estimation Methods

The SARS-CoV-2 cumulative prevalence was estimated from observed antibody reactivity using two sequential steps: (1) post-stratification weighting to standardize to the Texas population and (2) adjustment by antibody test sensitivity and specificity. First, crude observed seroprevalence was adjusted by age- and sex using weights derived from the U.S. census population projections for the state of Texas. Age in years was categorized into four categories: 18–34 years, 35–44 years, 45–54 years, and 55 years or greater. Post-stratification weights were computed to standardize our sample to the greater Texas population according to the 2019 projected census; the weight was computed as a ratio of the proportion of a given level of a stratum in the census, divided by the equivalent proportion in the sample. An adjustment for the assay sensitivity (99.82%) and specificity (99.91%) was applied as per Royal and colleagues. The full adjustment analysis was completed using IBM^®^ Statistical Package for the Social Science (SPSS^®^) Statistics Version 27 (United States) and by hand. The weights are then applied to the individuals in our data set using standard survey weighting methods. Finally, to adjust for assay characteristics, the cumulative adjusted prevalence is computed as per Rosenberg et al. (4):


$$\begin{array}{l} cumulative\;prevalence\;=\;\frac{proportion\;positive\;+\;specificity\;-1}{sensitivity\;+specificity\;-\;1}. \end{array}$$

Estimates that are age and sex-standardized and adjusted for test characteristics are henceforth called “fully adjusted estimates.” “Crude estimates” refer to the observed seroprevalence estimates.

## Results

### SARS-CoV-2 Antibodies Among Total Sample

Crude and adjusted SARS-CoV-2 antibody seropositivity are shown in Table 1. The crude antibody positivity prevalence in Phase I was 26.1% (80/307). The fully adjusted seroprevalence of the sample was 31.5%. Specifically, 41.1% of males and 21.9% of females were seropositive. For age categories, 33.5% of those 18–34; 24.4% of those 35–44; 33.2% of those 45–54; and 32.8% of those 55+ were seropositive.

**Table 1:** Seroprevalence rates by age and gender, standardized to Texas Census data and adjusted for assay characteristics.

		**Crude (observed) antibody N and %**				**TX census weighted N and %**					**Adjusted for assay (Spec** **=** **99.81%)**		
		**Antibody N**		**Antibody %**			**Weighted Antibody N**		**Weighted Antibody %**		**Adjusted antibody proportion**		
	**Phase I**	**Positive**	**Negative**	**Positive**	**Negative**	**Weighted**	**Positive**	**Negative**	**Positive**	**Negative**	**Positive**	**Estimated infection experienced**	**Proportion of estimated infection experienced**
Overall	307	80	227	26.1%	73.9%	307	97	210	31.6%	68.4%	31.5%	6795393	100%
**Sex**
Male	63	26	37	41.3%	58.7%	155	62	89	41.1%	58.9%	41.0%	4352534	64.1%
Female	243	54	189	22.2%	77.8%	152	35	121	22.4%	77.6%	22.3%	2444240	36.0%
**Age group**
18–34	90	24	66	26.7%	73.3%	98	33	65	33.7%	66.3%	33.5%	2378518	35.0%
35–44	69	14	55	20.3%	79.7%	58	14	43	24.6%	75.4%	24.4%	962657	14.2%
45–54	74	23	51	31.1%	68.9%	76	25	50	33.3%	66.7%	33.2%	1180189	17.4%
55+	74	19	55	25.7%	74.3%	76	24	51	32.0%	68.0%	31.9%	2233957	32.9%

### Demographic and Clinical Correlates of Seropositivity

Demographic and clinical characteristics, by SARS-CoV-2 antibody seropositivity are presented for the total Phase I sample, FQHC clinical staff, and FQHC patient population in Table 2. As shown in Table 2, 17.7% (14/79) FQHC employees tested were positive and 27.9% (57/204) of FQHC patients were positive. The mean age of the entire sample (*N* = 307) was 43.7 (SD = 13.5). The group was primarily female (79%, *n* = 252), white (95.3%, *n* = 286), and of Hispanic ethnicity (81.7%, *n* = 255), with 8.7% (*n* = 25) having some high school or less and 19.8% (*n* = 57) having an advanced professional or academic degree. A total of 78% (*n* = 221)] was employed full-time and 79% (*n* = 228) reported having some type of health insurance. The clinical characteristics of the sample indicate that 27.7% (*n* = 78) were overweight and 59.9% (*n* = 169) were obese with the mean BMI = 32.6 (SD = 7.6). The majority of participants reported not using tobacco products in the past 2 weeks (88.7%, *n* = 260) and did not report use of vaping products in the past 2 weeks (96.8%, *n* = 272).

**Table 2:** Demographics and clinical characteristics, TX CARES, all phase 1 participants, 2020.

**Demographics**	Overall (*n* = 319)^*^	**SARS-CoV2 antibody status**	
		**Positive (*n* = 80)**	**Negative (*n* = 227)**
	**N (%)**	**N (%)**	**N (%)**
**Cohort (*****n*** **=** **291)**			
FQHC Employee	82 (25.7)	14 (17.5)	65 (28.6)
FQHC Patient	209 (65.5)	57 (71.3)	147 (64.8)
Missing	28 (8.8)	9 (11.3)	15 (6.6)
**Gender (*****n*** **=** **319)**			
Male	67 (21.0)	26 (32.5)	38 (16.7)
Female	252 (79.0)	54 (67.5)	189 (83.3)
Missing	0 (0.0)	0 (0.0)	0 (0.0)
**Race (*****n*** **=** **300)**			
White	286 (89.7)	73 (91.2)	202 (89.0)
Black/African American	5 (1.6)	0 (0.0)	5 (2.2)
Asian	3 (0.9)	1 (1.2)	2 (0.9)
Hawaiian or Other Pacific Islander	1 (0.3)	0 (0.0)	1 (0.4)
American Indian or Alaska Native	2 (0.6)	0 (0.0)	1 (0.4)
Multi-racial	3 (0.9)	1 (1.2)	2 (0.9)
Missing	19 (6.0)	5 (6.2)	14 (6.2)
**Hispanic Ethnicity (*****n*** **=** **312)**			
Yes	255 (79.9)	75 (93.8)	171 (75.3)
No	57 (17.9)	3 (3.8)	51 (22.5)
Missing	7 (2.2)	2 (2.5)	5 (2.2)
**Education (*****n*** **=** **288)**			
Less than high school	12 (3.8)	6 (7.5)	6 (2.6)
Some high school	13 (4.1)	4 (5.0)	9 (4.0)
High school graduate/GED	78 (24.5)	27 (33.8)	48 (21.1)
Some college, no degree	68 (21.3)	20 (25.0)	48 (21.1)
Two- or four-year college degree	60 (18.8)	12 (15.0)	46 (20.3)
Advanced professional or academic degree	57 (17.9)	4 (5.0)	50 (22.0)
Missing	31 (9.7)	7 (8.8)	20 (8.8)
**Employment (*****n*** **=** **283)**			
Full-time	221 (69.3)	39 (48.8)	175 (77.1)
Part-time	12 (3.8)	6 (7.5)	6 (2.6)
Unemployed	38 (11.9)	19 (23.8)	18 (7.9)
Other	12 (3.8)	5 (6.2)	7 (3.1)
Missing	36 (11.3)	11 (13.8)	21 (9.3)
**Has Insurance (*****n*** **=** **289)**			
Yes	228 (71.5)	40 (50.0)	181 (79.7)
No	61 (19.1)	30 (47.5)	30 (13.2)
Missing	30 (9.4)	10 (12.5)	16 (7.0)
**BMI, categorical (*****n*** **=** **282)**			
Underweight	3 (0.9)	2 (2.5)	1 (0.4)
Normal	32 (10.0)	6 (7.5)	23 (10.1)
Overweight	78 (24.5)	21 (26.2)	56 (24.7)
Obese	169 (53.0)	41 (51.2)	124 (54.6)
Missing	37 (11.6)	10 (12.5)	23 (10.1)
**Frequency of smoking or use of other tobacco products in past 2 weeks**			
**(*****n*** **=** **293)**			
Not at all	260 (81.5)	67 (83.8)	186 (81.9)
Rarely	8 (2.5)	2 (2.5)	6 (2.6)
Once a day	9 (2.8)	3 (3.8)	5 (2.2)
More than once a day	16 (5.0)	1 (1.2)	15 (6.6)
Missing	26 (8.2)	7 (8.8)	15 (6.6)
**Frequency of using vaping products in past 2 weeks (*****n*** **=** **281)**			
Not at all	272 (85.3)	69 (86.2)	195 (85.9)
Rarely	6 (1.9)	0 (0.0)	6 (2.6)
Once a day	0 (0.0)	0 (0.0)	0 (0.0)
More than once a day	3 (0.9)	1 (1.2)	2 (0.9)
Missing	38 (11.9)	10 (12.5)	24 (10.6)
	**Mean (±SD)**	**Mean (±SD)**	**Mean (±SD)**
Age, years (*n* = 317; missing = 2)	43.7 (13.5)	43.3 (13.8)	43.6 (13.4)
Height, inches (*n* = 287; missing = 32)	64.2 (3.8)	64.6 (4.5)	64.0 (3.6)
Weight, pounds (*n* = 288; missing = 31)	191.4 (46.9)	188.1 (43.5)	192.0 (47.3)
BMI, continuous (*n* = 281; missing = 38)	32.6 (7.6)	31.6 (6.8)	33.0 (7.8)

* *Twelve individuals completed a survey, but did not complete an antibody test*.

### SARS-CoV-2 Symptoms and Previous Diagnoses

In Table 3, of those 80 people with a positive SARS-CoV-2 antibody test 78.9% (56/71) reported having had at least one symptom of COVID-19. Of those 227 who were negative, 38% (71/186) reported presence of COVID-19 symptoms. More than half (53.1%, 154/290) of the participants reported having had a previous COVID-19 test. Of the 154, 152 responded whether that test was positive or negative: 61/152 indicated it was positive (40.1%). In this sample, 42.2% (89/211) of those negative for the antibody test reported having had a COVID-19 test.

**Table 3:** SARS-CoV2 symptoms and previous diagnosis, TX CARES, all phase 1 participants, 2020.

**Previous COVID-19 diagnosis/symptoms**	Overall (*n* = 319)^*^	**SARS-CoV2 antibody status**	
		**Positive (*n* = 80)**	**Negative (*n* = 227)**
	**N (%)**	**N (%)**	**N (%)**
**Any COVID-19 Symptoms (*****n*** **=** **265)**			
Yes	130 (40.8)	56 (70.0)	71 (31.3)
No	135 (42.3)	15 (18.8)	115 (50.7)
Missing	54 (16.9)	9 (11.3)	41 (18.1)
Previous COVID-19 test (*n* = 290)
Yes	154 (48.3)	62 (77.5)	89 (39.2)
No	136 (42.6)	10 (12.5)	122 (53.7)
Missing	29 (9.1)	8 (10.0)	16 (7.0)
**Previous positive COVID-19 test result (*****n*** **=** **152)**			
Yes	61 (19.1)	55 (68.8)	6 (2.6)
No	91 (28.5)	6 (7.5)	82 (36.1)
Missing	167 (52.4)	19 (23.8)	139 (61.2)
**Diagnosed with COVID-19 by health professional without test (*****n*** **=** **290)**			
Yes	11 (3.4)	7 (8.8)	3 (1.3)
No	279 (87.5)	65 (81.2)	207 (91.2)
Missing	29 (9.1)	4 (33.3)	17 (7.5)
	**Median (IQR)**	**Median (IQR)**	**Median (IQR)**
Symptoms^**^			
Fever or Chills (*n* = 68)	3.00 (2.00–4.00)	3.00 (2.00–4.00)	3.00 (2.50–4.00)
Missing n (%)	253 (79.3)	51 (63.8)	192 (84.6)
Cough (*n* = 71)	3.00 (2.00–4.00)	3.00 (2.00–4.00)	3.00 (2.00–4.00)
Missing n (%)	249 (78.1)	48 (60.0)	190 (83.7)
Shortness of breath/difficulty breathing (*n* = 53)	3.00 (2.00–4.00)	3.50 (3.00–4.75)	3.00 (2.00–4.00)
Missing *n* (%)	266 (83.4)	50 (62.5)	206 (90.7)
Fatigue (*n* = 79)	4.00 (3.00–4.00)	4.00 (3.00–5.00)	3.00 (3.00–4.00)
Missing n (%)	242 (75.9)	47 (58.8)	185 (81.5)
Muscle or body aches (*n* = 72)	3.00 (3.00–4.00)	4.00 (3.00–4.75)	3.00 (3.00–4.00)
Missing n (%)	248 (77.7)	46 (57.5)	192 (84.6)
Headaches (*n* = 83)	4.00 (3.00–5.00)	4.00 (3.00–5.00)	3.00 (3.00–4.00)
Missing n (%)	237 (74.3)	46 (57.5)	181 (79.7)
Congestion or runny nose (n = 63)	3.00 (2.00–4.00)	3.00 (2.00–4.00)	3.00 (2.00–4.00)
Missing n (%)	257 (80.6)	55 (68.8)	190 (83.7)
Diarrhea (*n* = 44)	3.00 (2.00–3.00)	3.00 (2.00–4.00)	3.00 (2.00–3.00)
Missing n (%)	276 (86.5)	60 (75.0)	204 (89.9)
Nausea or vomiting (n = 37)	3.00 (2.00–4.00)	3.00 (2.00–5.00)	3.00 (2.00–4.00)
Missing n (%)	282 (88.4)	63 (78.8)	207 (91.2)
New loss of taste or smell (*n* = 44)	5.00 (4.00–5.00)	5.00 (4.00–5.00)	4.00 (3.50–5.00)
Missing n (%)	276 (86.5)	45 (56.3)	220 (96.9)
Sore throat (*n* = 68)	3.00 (2.00–4.00)	3.00 (2.00–4.00)	3.00 (2.00–3.25)
Missing n (%)	253 (79.3)	55 (68.8)	187 (82.4)

* *Twelve individuals completed a survey, but did not complete an antibody test*.

** *Symptom severity based on a scale from 1 to 5 with 1 being minimal and 5 being severe*.

Of the 61 respondents with a prior positive COVID-19 test, 55 (90.2%) had antibodies and 6 (9.8%) did not have antibodies. Of those diagnosed with COVID-19 by a health professional without a test, 7 (70.0%) had a positive antibody test and 3 (30%) had a negative antibody test result. The most commonly reported symptoms in the sample positive for SARS-CoV-2 antibodies were new loss of taste or smell, fatigue, muscle or body aches, and headaches.

#### Presence of Chronic Diseases in the Texas CARES Phase I Sample

From Table 4, those with a positive SARS-CoV-2 antibody test were most likely to report having the following chronic diseases: hypertension (21/68, 30.9%), diabetes (18/68, 26.5%), asthma (18/68, 26.5%), and obesity (14/68, 20.6%).

**Table 4:** Chronic diseases, TX CARES, all phase 1 participants, 2020.

**Chronic disease**	**Overall (*n* = 270)**	**SARS-CoV2 antibody status**	
		**Positive (*n* = 68)**	**Negative (*n* = 193)**
	**N (%)**	**N (%)**	**N (%)**
Asthma	56 (20.7)	18 (26.5)	33 (17.1)
COPD	4 (1.5)	1 (1.5)	2 (1.0)
Cancer	7 (2.6)	0 (0.0)	7 (3.6)
Cardiovascular	8 (3.0)	2 (2.9)	5 (2.6)
Diabetes	56 (20.7)	18 (26.5)	35 (18.1)
Hypertension	83 (30.7)	21 (30.9)	58 (30.1)
Obesity	60 (22.2)	14 (20.6)	43 (22.3)
Sickle cell	0 (0.0)	0 (0.0)	0 (0.0)
Immunocompromised	8 (3.0)	1 (1.5)	6 (3.1)
Kidney disease	2 (0.7)	0 (0.0)	2 (1.0)
Other	17 (6.3)	2 (2.9)	13 (6.7)

## Discussion

In this study we enrolled and analyzed 307 participants, demonstrating a high survey and antibody test completion rate, and ability to implement a questionnaire and SARS-CoV-2 antibody testing within FQHC clinical settings. We were also able to determine our capability to estimate the cross-sectional seroprevalence within Texas's FQHC clinical settings. The crude positivity prevalence for SARS-CoV-2 antibodies in this sample was 26.1% indicating potentially high exposure to COVID-19 for FQHC clinic employees and patients. We also demonstrated feasibility and capability to determine the presence of IgG antibodies to SARS-CoV-2 in populations with and without previous COVID-19 positive diagnosis.

The inclusion of COVID-19 positive and negative participants is important as it has been a limitation of other studies and allows us to more accurately determine the seroprevalence and human response over time in a diverse representative population. Therefore, ability to determine antibodies in individuals with no previous history of COVID-19 over time is a unique aspect of our program approach that may inform understanding of the timing of neutralizing antibodies across a 6-month period; current estimate indicate antibodies may be stable for 5–7 months after SARS-CoV-2 infection (13). Ongoing analysis is focused on determining the time of contracting COVID-19 infection, antibody test and response over time with preliminary findings noting natural antibody levels may peak at 120 days with natural antibody test response lasting 200–500 days. Analysis to be reported elsewhere with oral presentation to the American Public Health Association, October 25, 2021.

Although self-reported the COVID-19 test positivity and self-report of symptoms allows us to better determine the cycle and decline of antibody levels in a large sample of Texans over a 6-month period. It is estimated that over one-third of patients that have recovered from COVID-19 have antibodies given mild or asymptomatic disease (11), it is important to note that in our sample, 68.7% of those with a previous positive COVID-19 test had a positive SARS-CoV-2 antibody test. As with other research we found that the links with BMI, previous history of chronic illness and age (2–9) were correlated to human response in this sample. It is also important that as public health practitioners we understand the impact of co-morbidities and different needs of populations and how demographics, behavioral and social variables impacted antibody response over time.

The timing of the data collection from the start of the first reported cases in Texas was ~6-months from the start of our data collection. The positive cases will be monitored for decline of antibody levels and collection of additional COVID-19 testing, positive results and symptoms over a further 6-month enrollment period. Although the highest neutralizing antibody titers are found in severe disease (19), the expected waning of antibody presence is yet unknown. We posit that the presence of antibodies will vary by populations, previous exposures and symptoms. The design of our program allows us to collect survey data to best identify the demographic and clinical characteristics associated with seroprevalence response across a large state. It is estimated that there may be 10 times more SARS-CoV-2 infections than the number of reported cases (14). Understanding the presence of antibodies in a large sample of diverse populations with and without COVID-19 diagnosis may also be used to inform state-wide initiatives, vaccination distribution and restrictions across large populations.

The Phase I setting is important to consider as we enrolled FQHCs to participate in the program to determine the presence of antibodies in both employees and patient populations. Given the predicted long-term health consequences of COVID-19 (19) the Texas CARES program focuses on reach of populations that are underinsured and likely to have co-morbid chronic conditions. The inclusion of this population will allow for the identification of percentage of high-risk patients with antibodies, informing their long-term care for cardiovascular, pulmonary, neurologic and emotional well-being. These data will allow for informed planning by FQHCs and state leaders to determine and address vulnerable patient population needs and for the development of interventions and strategies to best care to mitigate poor health effects of COVID-19 over time.

Among this sample, we found that our adjustments indicate that male patients may have a higher proportion of positivity for antibodies, likely due to greater exposure to COVID-19 by industry and continuation of work during restriction periods. Although the male and female sample sizes are unequal we adjusted to the Texas Census population allowing us to estimate the adjusted human response. This finding aligns with positive proportions of COVID-19 found in males as well as lower antibody levels found in women (30). Additionally, it is important to note the successful reach and high survey completion rate as a result of our engagement and communication strategies designed using a participatory approach to support community-academic partnerships. The engagement of FQHCs who primarily serve vulnerable populations disproportionately impacted by COVID-19 was purposeful as it allowed for reach and determination of antibody response in highly vulnerable patients.

This report has several limitations. First, the participants are voluntary and are not a representative sample of Texas residents. However, the sample represents patients and populations in three counties and areas with varied COVID-19 infection rates. Second, the data collection for COVID-19 test positivity are self-reported, however, we believe the pandemic and impact on communities increases reliability of self-reported testing and positive diagnosis. We have considered false-positive and false-negative results in analysis and are working on analysis to ensure that responses are better understood. As the TX CARES sample increases we hypothesize the prevalence of antibody positive will decrease as the Phase I population represented three specific FQHC clinic settings and communities. Third, this sample was primarily women, representing the employee demographic of FQHCs and patient populations within the clinics. Nevertheless, these findings suggest the feasibility to recruit participants from high-risk populations seeking care at FQHCs and employees serving the population. We also found that the high proportions of survey completion point to interest in the population to engage in research to identify antibody status.

## Conclusions

This program was designed to identify the humoral immune response to SARS-CoV-2 infection in a large sample over time and may assist in determining potential vulnerability to a surge in COVID-19 cases across a large state population. We found a high estimation of seroprevalence in this first phase of our program using a high specificity and sensitivity assay in a primarily White Hispanic population. Estimating seroprevalence is important given the potential for reinfection and severity of COVID-19 in vulnerable populations with co-morbidities while vaccination uptake and reach across a state continues.

As part of this first phase we have worked to enroll, reach and include vulnerable populations in antibody surveys to identify antibody response. Our additional analysis is now focused on identifying natural human response as well as vaccine induced response over time (6-months). This is important as public health must better understand the response over time and how long immunity may last. The Texas CARES program is collecting follow-up antibody testing data and behavioral, social and illness questionnaires to further identify not only natural human response but vaccine induced response and long term COVID-19 impact on chronic disease management in vulnerable populations, to date we have enrolled over 2,800 participants from FQHCs across Texas.

## What Is Already Known on This Topic?

Infection rates of SARS CoV-2 are documented across the world, however, estimates of true infection and “natural” immunity are still unclear. It is also important to understand the human response in vulnerable populations and those that serve them at community clinics.

## What Is Added by This Report?

This survey allows us to better understand “natural” immunity and exposure in a underserved population receiving care at Federally Qualified Health Centers (FQHCs) across the state of Texas. TX CARES also contributes to our understanding of engagement of underserved communities using strategies such as champions at the FQHC sites.

## What are the Implications for Public Health Practice?

Implications of this work include greater understanding of seroprevalence response as well as exposure across ages 5–80 years at FQHCs. Estimating seroprevalence is important for public health practices given the potential for reinfection and severity of COVID-19 in vulnerable populations with co-morbidities while vaccination of a larger portion of the population continues.

## Data Availability Statement

The raw data supporting the conclusions of this article will be made available by the authors, without undue reservation.

## Ethics Statement

The studies involving human participants were reviewed and approved by University of Texas Health Science Center in Houston Institutional Review Board. Written informed consent to participate in this study was provided by the participants' legal guardian/next of kin.

## Author Contributions

EB, DL, MS, JS, SP, and MV-S were responsible for the conception and design of the survey. MV-S led the relationship with regional FQHCs. MS, SD, AY, and LW lead the data coordination components including survey operation, including the coordination of data acquisition and logistics. MV-S and SM developed the operational protocols for field work and were responsible for training the involved administrative and health personnel. MV-S, AY, LW, MG, MS, and SD were in charge of statistical analyses and table and figure design. All remaining authors in the TX CARES group contributed to participant recruitment, data acquisition, laboratory analyses, and quality control for their respective populations. The first draft was written by MV-S and SD. All authors had full access to all study data, contributed to data interpretation, critically reviewed the first draft, approved the final version, and agreed to be accountable for the work.

## Funding

This work was supported by the Texas Department of State Health Services and the University of Texas System.

## Conflict of Interest

The authors declare that the research was conducted in the absence of any commercial or financial relationships that could be construed as a potential conflict of interest.

## Publisher's Note

All claims expressed in this article are solely those of the authors and do not necessarily represent those of their affiliated organizations, or those of the publisher, the editors and the reviewers. Any product that may be evaluated in this article, or claim that may be made by its manufacturer, is not guaranteed or endorsed by the publisher.
